# Assessment of myocardial perfusion-CMR in left main stem disease (LMS) in the CEMARC study

**DOI:** 10.1186/1532-429X-13-S1-P170

**Published:** 2011-02-02

**Authors:** Arshad Zaman, David L Buckley, Steven Sourbron, Neil Maredia, John F Younger, Julia M Brown, Jane Nixon, Colin C Everett, John P Ridgway, Aleksandra Radjenovic, Catherine J Dickinson, Mark Sculpher, Steven G Ball, John P Greenwood, Sven Plein

**Affiliations:** 1 Medical Physics, Leeds, UK; 2 Academic Unit of Cardiovascular Medicine, Leeds, UK; 3 Clinical Trials Research Unit, Leeds, UK; 4 Department of Nuclear Cardiology, Leeds, UK; 5 Centre for Health Economics, York, UK

## Introduction

Left main stem (LMS) disease is found in approximately 5% of patients with stable angina and in approximately 7% of patients presenting with an acute myocardial infarction. Accurate assessment of the degree of left main stem stenosis has important prognostic and therapeutic implications. Clinically, angiographic LMS stenosis of 50% or more is considered significant. However, it is not known how accurately myocardial perfusion imaging detects LMS disease at this severity threshold.

## Purpose

1. To measure myocardial blood flow by CMR in patients with LMS stenosis of more than 50% on quantitative angiography in the CEMARC study (a large prospective evaluation of CMR against SPECT and coronary angiography^1^).

2. To correlate hyperaemic myocardial blood flow (MBF) and blood flow reserve between territories supplied by the LMS and remote territories.

## Methods

Nine patients from the CEMARC study who were found to have significant LMS disease on quantitative coronary angiography underwent perfusion-CMR on a Philips 1.5 T Intera system. Myocaridal perfusion imaging was performed every heartbeat during the first pass of 0.05 mmol/kg gadolinium chelate using a T1-weighted fast (spoiled) GE sequence. Stress perfusion imaging was performed using intravenous adenosine infused for 4 minutes (140mcg/kg/min). Perfusion-CMR data were post-processed off-line using the software PMI^2^. Following motion correction a circular ROI was selected in the left ventricle to measure the arterial input function. MBF maps were created by model-free analysis; myocardial ROIs were drawn on these maps, one in the LMS territory (ROI1: LAD+LCX) and one in a remote region (ROI2:RCA). MBF for these ROIs was calculated using the Fermi model^3^. Statistical calculations were performed using SPSS.

## Results

**Figure 1:**
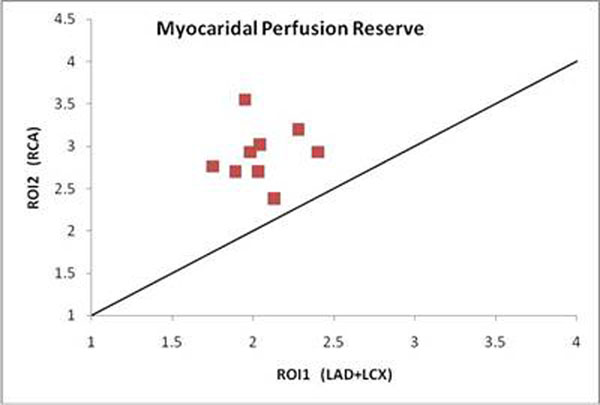
MPR values in LMS diseased terotories

Of the 9 datasets analysed, the results revealed significant differences (p<0.001) in myocardial perfusion seen in LMS diseased territories (ROI1) compared to normal segments (ROI2), Figure 1. The mean myocardial perfusion reserve (MPR) of ROI1 was 2.05 (SD ±0.20) and for ROI2 2.97 (±0.33).

## Conclusion

This study demonstrates reduced myocardial blood flow reserve in patients with LMS stenosis of 50% or more, although reductions are subtle.
